# Circadian Blood Pressure Patterns in Adults with Moderate-to-Severe Hepatic Steatosis: A Prospective Ambulatory Blood Pressure Monitoring Study

**DOI:** 10.3390/jcm15155988

**Published:** 2026-08-01

**Authors:** Nurcan Aslan, Mustafa Comoglu, Emra Asfuroglu Kalkan, Oguzhan Zengin, Huseyin Camli, Ihsan Ates

**Affiliations:** Department of Internal Medicine, Ankara Bilkent City Hospital, 06800 Ankara, Türkiye; drnurcanaslan@gmail.com (N.A.); emrasfur@yahoo.com (E.A.K.); oguzhanzengin91@gmail.com (O.Z.); drhuseyincamli@gmail.com (H.C.); dr.ihsanates@hotmail.com (I.A.)

**Keywords:** hepatic steatosis, ambulatory blood pressure monitoring, circadian blood pressure, nocturnal blood pressure, non-dipping blood pressure

## Abstract

**Objective:** Hepatic steatosis commonly coexists with cardiometabolic risk factors, yet circadian blood pressure patterns in adults with hepatic steatosis remain incompletely characterized. This study aimed to describe ambulatory blood pressure patterns in adults with ultrasonographically detected moderate-to-severe hepatic steatosis. **Methods:** This single-center prospective observational study enrolled adults with grade 2 or 3 hepatic steatosis on abdominal ultrasonography between August 2023 and August 2024. All participants underwent 24-h ambulatory blood pressure monitoring after enrollment. Patients were classified as dipper or non-dipper according to nocturnal blood pressure decline, and clinical, biochemical, ultrasonographic, and ambulatory parameters were compared. **Results:** Among 137 patients, 43 (31.4%) had a dipper pattern and 94 (68.6%) had a non-dipper pattern. Grade 2 and grade 3 steatosis were present in 125 (91.2%) and 12 (8.8%) patients, respectively. In the exploratory multivariable analysis, age, sex, antihypertensive treatment, and ultrasonographic steatosis grade were not independently associated with non-dipping status. Ambulatory hypertension thresholds were met more often in the non-dipper group [72 (76.6%) vs. 25 (58.1%), *p* = 0.027]. Nocturnal hypertension was more frequent in the non-dipper group [72 (76.6%) vs. 22 (51.2%), *p* = 0.003]. The non-dipper group had higher 24-h systolic blood pressure [134.0 (122.0–145.0) vs. 130.0 (118.5–136.0) mmHg, *p* = 0.027]. The median nocturnal systolic blood pressure decline in the overall cohort was 6.0% [2.6–11.3], whereas daytime systolic and diastolic blood pressure values were comparable between groups. **Conclusions:** Non-dipping blood pressure was frequent in adults with moderate-to-severe hepatic steatosis. Ambulatory blood pressure monitoring provides a descriptive assessment of nocturnal blood pressure patterns in this selected hepatic steatosis cohort.

## 1. Introduction

Hepatic steatosis frequently coexists with obesity, dyslipidemia, impaired glucose metabolism, and hypertension [1]. This metabolic overlap is clinically relevant because cardiovascular disease contributes substantially to long-term morbidity and mortality in populations with hepatic steatosis [2].

Blood pressure regulation is one of the most accessible markers of vascular risk in this setting. Although measuring blood pressure in the office remains central to clinical practice, it cannot fully characterize nocturnal hemodynamic behavior. Ambulatory blood pressure monitoring provides information on daytime and nighttime blood pressure, nocturnal hypertension, and the expected physiological decline in blood pressure during sleep [3]. A nocturnal fall of at least 10% defines the dipper pattern, whereas a blunted fall defines the non-dipper pattern [4]. Non-dipping blood pressure has been linked to target-organ damage and adverse cardiovascular outcomes independently of traditional blood pressure categories [5].

Several mechanisms have been proposed to explain why hepatic steatosis and altered circadian blood pressure patterns may coexist in cardiometabolic populations. Hepatic steatosis has been linked to endothelial dysfunction, oxidative stress, insulin resistance, and altered adipokine signaling, all of which may influence vascular tone and nocturnal blood pressure regulation [6,7]. Previous studies using nonalcoholic fatty liver disease criteria have reported a higher prevalence of hepatic steatosis, lower adiponectin levels, and greater insulin resistance among non-dipper hypertensive patients [8]. Other studies have also suggested that altered dipping patterns tend to coexist with unfavorable metabolic and lipid profiles [9,10].

Against this background, characterization of nocturnal blood pressure behavior in patients with clinically evident hepatic steatosis may provide descriptive information on circadian blood pressure patterns in this population. Therefore, this study aimed to describe ambulatory blood pressure patterns in adults with ultrasonographically detected moderate-to-severe hepatic steatosis and to compare their clinical, biochemical, ultrasonographic, and ambulatory characteristics according to dipping status.

## 2. Materials and Methods

### 2.1. Study Design and Population

This single-center prospective observational study was conducted in the Department of Internal Medicine at Ankara Bilkent City Hospital between August 2023 and August 2024. The study protocol was approved by the Ankara Bilkent City Hospital Non-Interventional Clinical Research Ethics Committee on 9 August 2023 (approval number: E2-23-4607). The study was conducted in accordance with the principles of the Declaration of Helsinki, and written informed consent was obtained from all participants before 24-h ambulatory blood pressure monitoring. This report was prepared in accordance with the Strengthening the Reporting of Observational Studies in Epidemiology (STROBE) statement.

Adult patients evaluated in the outpatient clinics of the Department of Internal Medicine who were found to have grade 2 or grade 3 hepatic steatosis on routine abdominal ultrasonography [11] were consecutively assessed for eligibility. Eligible patients who met the predefined inclusion and exclusion criteria were enrolled. Patients with incomplete clinical or laboratory data, lack of regular follow-up data, known chronic liver disease other than hepatic steatosis, chronic kidney disease, coronary artery disease, or diabetes mellitus were excluded. Patients with coronary artery disease were excluded to reduce the potential influence of established macrovascular disease and related cardiovascular treatment on ambulatory blood pressure patterns. After enrollment, all participants underwent prospective 24-h ambulatory blood pressure monitoring, and patients were subsequently categorized as having a dipper or non-dipper pattern according to the monitoring results.

Participants were enrolled on the basis of grade 2 or grade 3 hepatic steatosis detected by ultrasonography rather than according to formal diagnostic criteria for metabolic dysfunction-associated steatotic liver disease. Alcohol intake was not systematically collected or quantified; therefore, participants could not be classified into MASLD, MetALD, or other etiologic subtypes of steatotic liver disease. The study protocol did not include dedicated assessment for the lean MASLD phenotype, MASH, or hepatic fibrosis. Consequently, the presence of MASH and the presence or stage of hepatic fibrosis could not be established, although patients with known chronic liver diseases other than hepatic steatosis were excluded.

### 2.2. Definitions and Data Collection

Clinical and laboratory data were obtained from the hospital electronic medical record system. Age, sex, comorbid conditions, medication history, and baseline vital signs were recorded. Laboratory variables included fasting plasma glucose, urea, creatinine, electrolytes, albumin, liver enzymes, bilirubin levels, complete blood count parameters, triglycerides, total cholesterol, low-density lipoprotein cholesterol, and high-density lipoprotein cholesterol.

Hepatic steatosis was assessed by routine abdominal or hepatobiliary ultrasonography performed within the 3 months preceding ambulatory blood pressure monitoring. Grade 2 steatosis was defined by a moderate diffuse increase in hepatic echogenicity with reduced visualization of the intrahepatic vessels and diaphragm, whereas grade 3 steatosis was defined by marked diffuse echogenicity, impaired visualization of the intrahepatic vessels and diaphragm, and poor penetration of the posterior liver segments [11]. Ultrasonographic examinations were performed by radiologists who were unaware of the subsequent ambulatory blood pressure monitoring results. Formal interobserver reliability was not assessed. Patients with grade 2 or grade 3 hepatic steatosis were included, and ultrasonographic steatosis grade was used for descriptive subgroup comparisons. No additional imaging modality or liver biopsy was performed as part of the study protocol. The main clinical grouping variable was circadian blood pressure pattern, classified as dipper or non-dipper according to 24-h ambulatory blood pressure monitoring. Additional comparisons were performed according to ultrasonographic steatosis grade and ambulatory hypertension status.

### 2.3. Ambulatory Blood Pressure Monitoring

After enrollment, all participants underwent 24-h ambulatory blood pressure monitoring using a SunTech Bravo device. An appropriately sized cuff was placed on the upper arm, and the device was programmed to obtain measurements every 20 min during wakefulness and every 30 min during sleep. Participants were asked about their usual sleep and wake times before monitoring, and the daytime and nighttime periods were individually programmed into the device according to these reported times. They were instructed to continue their usual daily activities, avoid excessive physical exertion, and keep the monitored arm still during measurements.

A recording was considered technically valid when at least 70% of the programmed measurements were successful, including at least 20 valid daytime readings and 7 valid nighttime readings. Ambulatory blood pressure monitoring was repeated when these criteria were not met. Ambulatory hypertension was defined according to the 2023 European Society of Hypertension guideline thresholds as a 24-h mean blood pressure ≥ 130/80 mmHg, daytime mean blood pressure ≥135/85 mmHg, or nighttime mean blood pressure ≥ 120/70 mmHg [3]. Isolated daytime hypertension was defined as elevated daytime blood pressure with normal nighttime blood pressure, isolated nocturnal hypertension as elevated nighttime blood pressure with normal daytime blood pressure, and sustained ambulatory hypertension as elevated blood pressure during both daytime and nighttime periods. Classification was based exclusively on the measured ambulatory blood pressure values, irrespective of known hypertension or antihypertensive treatment status. Antihypertensive treatment was recorded separately as medication use at the time of enrollment.

The percentage nocturnal systolic blood pressure decline was calculated as follows: [(mean daytime systolic blood pressure − mean nighttime systolic blood pressure)/mean daytime systolic blood pressure] × 100. Dipping status was determined according to the nocturnal systolic blood pressure decline. A decline of ≥10% was classified as a dipper pattern, whereas a decline of <10% was classified as a non-dipper pattern. Diastolic blood pressure decline was not used to determine the primary classification; therefore, discordance between systolic and diastolic dipping did not alter group assignment. Reverse-dipper and extreme-dipper patterns were not evaluated as separate categories in the original study protocol.

#### Sample Size Calculation

Based on previous reports indicating that non-dipping blood pressure patterns occur in approximately 40–60% of hypertensive and metabolically at-risk populations [9,12], the expected prevalence was set at 50%, representing the most conservative assumption for sample-size estimation. With a 95% confidence level and an absolute precision of 9%, the minimum required sample size was calculated as 119 participants. Allowing for approximately 10% technically invalid ambulatory blood pressure recordings or incomplete data, the target sample size was set at 132 participants. The final study cohort included 137 participants.

### 2.4. Statistical Analysis

IBM SPSS Statistics version 27.0 was used to conduct statistical analysis. The Shapiro–Wilk test and visual inspection of histograms were used to assess the distribution of continuous variables. Depending on their distribution, continuous variables were presented as mean ± standard deviation or median with interquartile range values. Numbers and percentages were used to summarize categorical variables. The independent-samples t-test for normally distributed continuous variables and the Mann–Whitney U test for non-normally distributed variables were used to compare two groups. Categorical variables were compared using the Pearson chi-square test or Fisher’s exact test, as appropriate.

An exploratory multivariable binary logistic regression analysis was performed with non-dipping status as the dependent variable. Model complexity was limited in consideration of the sample size. The results are reported as odds ratios with 95% confidence intervals. Inferential comparisons were not performed for nocturnal systolic or diastolic blood pressure decline percentages because systolic decline defined the primary dipping classification and diastolic decline was a closely related derived measure. No formal adjustment for multiple comparisons was applied; therefore, isolated findings from secondary biochemical and heart-rate comparisons were interpreted as exploratory. Statistical significance was defined as a two-sided *p*-value of less than 0.05.

## 3. Results

A total of 137 patients with ultrasonographically detected grade 2 or higher hepatic steatosis were included in the study. Of these, 43 patients (31.4%) had a dipper blood pressure pattern and 94 patients (68.6%) had a non-dipper pattern. Grade 2 steatosis was present in 125 patients (91.2%), whereas 12 patients (8.8%) had grade 3 steatosis (Table 1). Among patients with grade 2 and grade 3 steatosis, dipper patterns were observed in 40 of 125 (32.0%) and 3 of 12 (25.0%) patients, respectively.

Baseline clinical and biochemical characteristics according to circadian blood pressure pattern were compared between dipper and non-dipper patients. The median age was 47.0 years in the dipper group and 51.0 years in the non-dipper group, with no significant difference between groups (*p* = 0.308). Male sex was also comparable between the groups [26 (60.5%) vs. 47 (50.0%), *p* = 0.255]. Ambulatory hypertension was more frequent in the non-dipper group than in the dipper group [72 (76.6%) vs. 25 (58.1%), *p* = 0.027]. Antihypertensive medication use was similar between the groups [13 (30.2%) vs. 34 (36.2%), *p* = 0.564]. Ultrasonographic steatosis grade did not differ significantly according to circadian blood pressure pattern (*p* = 0.445) (Table 2).

Most biochemical parameters were comparable between patients with dipper and non-dipper patterns. Fasting plasma glucose, urea, creatinine, sodium, potassium, albumin, aminotransferases, triglycerides, total cholesterol, LDL cholesterol, and HDL cholesterol did not differ significantly between the groups. The only significant biochemical difference was observed in GGT levels, which were higher in the dipper group than in the non-dipper group [34.0 (26.0–58.5) vs. 28.5 (19.2–44.8) U/L, *p* = 0.023] (Table 2).

**Figure 1:**
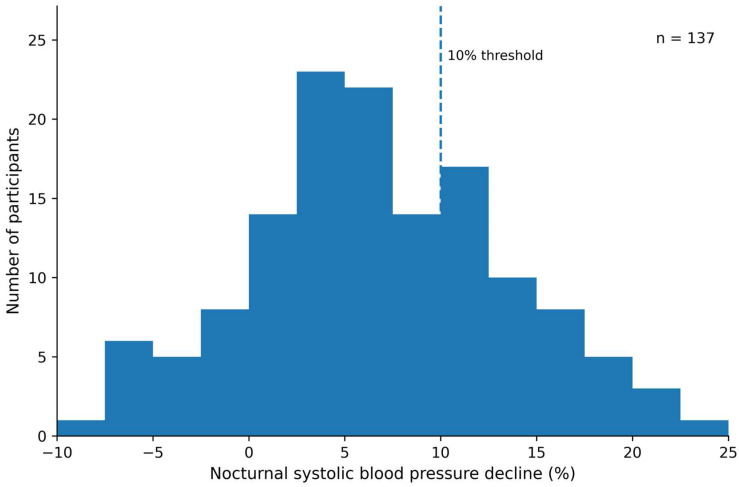
Distribution of nocturnal systolic blood pressure decline in the study cohort.

Ambulatory blood pressure monitoring showed a distinct nocturnal profile in patients with a non-dipper pattern. The 24-h mean systolic blood pressure was higher in the non-dipper group than in the dipper group [134.0 (122.0–145.0) vs. 130.0 (118.5–136.0) mmHg, *p* = 0.027], whereas 24-h mean diastolic blood pressure did not differ significantly (*p* = 0.335). Daytime systolic and diastolic blood pressures were also comparable between groups. In the overall cohort, the median nocturnal systolic and diastolic blood pressure declines were 6.0% [2.6–11.3] and 9.7% [5.0–16.9], respectively. Nocturnal hypertension was more frequent in the non-dipper group [72 (76.6%) vs. 22 (51.2%), *p* = 0.003]. In the overall cohort, isolated daytime hypertension was present in 3 patients (2.2%), isolated nocturnal hypertension in 20 (14.6%), and sustained ambulatory hypertension in 74 (54.0%); 40 participants (29.2%) met neither the daytime nor the nighttime hypertension threshold. As expected from the classification, nocturnal decline percentages were presented descriptively without inferential testing. The dipper group had higher 24-h mean heart rate and daytime mean heart rate, while nighttime mean heart rate was similar between the groups; these heart-rate differences were considered secondary exploratory findings (Table 3). The distribution of nocturnal systolic blood pressure decline is shown in Figure 1.

In the exploratory multivariable logistic regression analysis including age, sex, antihypertensive treatment, and ultrasonographic steatosis grade, none of the available covariates was independently associated with non-dipping status. Age, male sex, antihypertensive treatment, and grade 3 hepatic steatosis all had confidence intervals crossing unity (Table 4).

## 4. Discussion

The present study describes ambulatory blood pressure patterns in adults with ultrasonographically evident moderate-to-severe hepatic steatosis. More than two-thirds of participants exhibited a non-dipping pattern, and nocturnal hypertension was also common, despite broadly similar daytime systolic and diastolic blood pressure values across dipping groups. These findings suggest that daytime measurements may not fully capture circadian blood pressure behavior in this cohort. However, the absence of a non-steatotic comparison group precludes attributing the observed nocturnal patterns specifically to hepatic steatosis. Nevertheless, the findings provide a clinically informative characterization of nocturnal blood pressure behavior in this population.

Hepatic steatosis commonly occurs within a broader metabolic context characterized by insulin resistance, atherogenic dyslipidemia, systemic inflammation, endothelial injury, and altered lipid handling [13,14]. Contemporary reviews and clinical guidance emphasize the relevance of cardiovascular risk factors and associated metabolic comorbidities in patients with steatotic liver disease [15,16]. Nevertheless, the present design does not allow the observed frequency of non-dipping to be attributed either to hepatic fat accumulation or to the broader metabolic environment accompanying steatosis. The absence of marked biochemical differences between dipper and non-dipper patients should therefore be regarded as a descriptive finding rather than evidence for a specific underlying mechanism.

Previous ABPM studies have reported broadly similar patterns, although patient selection and the blood pressure phenotypes evaluated have varied considerably. Fallo et al. reported that fatty liver disease was more frequent among non-dipper than dipper patients with untreated essential hypertension, together with lower adiponectin levels and greater insulin resistance [8]. Latea et al. similarly found that fatty liver disease clustered with altered circadian blood pressure patterns in patients with severe hypertension [9]. In a retrospective ABPM cohort of 142 hypertensive and normotensive adults, Astan et al. observed greater ultrasonographic steatosis severity among patients with non-dipper hypertension and reported that steatosis grade discriminated between hypertensive dipper and non-dipper patterns with an AUC of 0.861 [17]. A separate study by Astan et al. involving patients without previously diagnosed hypertension also linked greater steatosis severity to an unfavorable nocturnal blood pressure phenotype [18]. Martin et al. extended this evidence to another ambulatory phenotype by reporting masked hypertension among patients with MASLD, highlighting abnormalities that may not be apparent from office blood pressure measurements alone [12]. Population-based data have likewise shown that non-dipping may coexist with obesity, adverse lipid parameters, and impaired glucose metabolism even outside established hypertension or diabetes [19]. Unlike these studies, our cohort was selected specifically on the basis of moderate-to-severe ultrasonographic hepatic steatosis and was analyzed primarily according to systolic dipping status. The available evidence therefore supports the value of ABPM for characterizing heterogeneous blood pressure phenotypes in populations with hepatic steatosis, but it does not establish an independent effect of hepatic steatosis on nocturnal blood pressure regulation.

Several overlapping mechanisms have been proposed to explain the coexistence of hepatic steatosis and non-dipping blood pressure in cardiometabolic populations. Endothelial dysfunction has been demonstrated in populations with hepatic steatosis and supported by pooled vascular data, but this is unlikely to act in isolation [6,7]. Adiponectin deficiency and insulin resistance are particularly relevant in this context: both have been linked to NAFLD, and lower adiponectin with greater insulin resistance has also been reported in non-dipper hypertensive patients [20,21]. Autonomic imbalance may provide another connection, as reduced parasympathetic activity and heightened sympathetic tone have been associated with fatty liver development and with impaired nocturnal blood pressure decline in metabolic syndrome [22,23]. In the present study, the routine biochemical profile did not sharply distinguish dipper from non-dipper patients, apart from higher GGT levels in the dipper group. Higher GGT levels and higher 24-h and daytime heart rates in the dipper group should be regarded as secondary exploratory findings, given the number of comparisons performed and the absence of formal multiplicity adjustment. Differences in physical activity, medication effects, autonomic function, or chance may have contributed to the heart-rate findings. The limited biochemical separation between groups may be partly related to the deliberate exclusion of patients with diabetes mellitus, coronary artery disease, and chronic kidney disease, which likely reduced the burden of overt metabolic and vascular disease in the cohort. However, because dipping status was defined according to nocturnal systolic blood pressure decline, the decline measures were reported descriptively without inferential testing, whereas nocturnal hypertension was evaluated separately using absolute nighttime blood pressure thresholds.

The exploratory adjusted analysis did not identify an independent association between non-dipping status and age, sex, antihypertensive treatment, or ultrasonographic steatosis grade. This result supports a cautious descriptive interpretation of the cohort but should not be regarded as evidence that these factors are unrelated to circadian blood pressure behavior, given the limited number of available covariates and the absence of several important anthropometric, sleep-related, and treatment-related variables.

Most patients in the cohort had grade 2 steatosis, and the number of patients with grade 3 steatosis was small. Therefore, the distribution of dipping patterns across steatosis grades should be interpreted descriptively. Conventional ultrasonographic grading may also be insufficient to reflect the vascular and metabolic heterogeneity of fatty liver disease.

From a clinical perspective, the frequent occurrence of non-dipping and nocturnal hypertension despite comparable daytime systolic and diastolic blood pressure values suggests that daytime measurements alone may not fully characterize circadian blood pressure behavior in adults with moderate-to-severe hepatic steatosis. When ABPM is otherwise clinically indicated, careful evaluation of nocturnal blood pressure may reveal an unfavorable nighttime phenotype and help identify individuals who warrant closer cardiovascular assessment. However, the present findings do not support routine ABPM screening solely on the basis of hepatic steatosis.

Several limitations should be considered when interpreting these findings. First, this was a single-center study without a control group lacking hepatic steatosis; therefore, the observed frequency of non-dipping cannot be compared with that in non-steatotic populations or attributed specifically to hepatic steatosis. Second, participants were enrolled on the basis of grade 2 or grade 3 ultrasonographic steatosis rather than formal MASLD criteria, and steatotic liver disease subtypes were not systematically classified. The time since the initial recognition of hepatic steatosis was not systematically recorded, and subtype-specific analyses could not be performed. Hepatic steatosis was graded by routine ultrasonography rather than magnetic resonance–based fat quantification, elastography, or histology, and therefore the presence of MASH, hepatic fibrosis, and fibrosis stage could not be determined. Formal interobserver reliability was not assessed, and the small number of participants with grade 3 steatosis limited comparisons according to steatosis severity. Finally, important determinants of nocturnal blood pressure, including body mass index, waist circumference, smoking, physical activity, and obstructive sleep apnea, were not systematically available; therefore, residual confounding cannot be excluded. Nevertheless, the prospective design and standardized acquisition of ambulatory blood pressure data strengthen the reliability of the circadian blood pressure classification.

In conclusion, non-dipping and nocturnal hypertension were common in adults with moderate-to-severe hepatic steatosis, despite broadly comparable daytime blood pressure values. These findings provide a clinically relevant characterization of nocturnal blood pressure behavior in this population. However, without a non-steatotic comparison group, they do not establish that hepatic steatosis is independently associated with non-dipping. Controlled studies incorporating appropriate comparison groups, validated liver phenotyping, and relevant metabolic, sleep-related, and treatment-related factors are needed to clarify this relationship.

## Figures and Tables

**Table 1:** Distribution of circadian blood pressure patterns in the study cohort.

Variable	Overall Cohort (*n* = 137)
*Circadian blood pressure pattern*	
Dipper pattern	43 (31.4)
Non-dipper pattern	94 (68.6)
Antihypertensive medication use	47 (34.3)
*Hepatic steatosis grade*	
Grade 2	125 (91.2)
Grade 3	12 (8.8)

Data are presented as number (%).

**Table 2:** Baseline clinical and biochemical characteristics according to circadian blood pressure pattern.

Variable	Dipper Pattern (*n* = 43)	Non-Dipper Pattern (*n* = 94)	*p* Value
Age, years	47 (40–56)	51 (40.2–57)	0.308
Male sex, *n* (%)	26 (60.5)	47 (50.0)	0.255
Ambulatory hypertension, *n* (%)	25 (58.1)	72 (76.6)	0.027
Antihypertensive medication use, *n* (%)	13 (30.2)	34 (36.2)	0.564
Antihypertensive regimen, *n* (% of treated patients)			0.739
Monotherapy	4 (30.8)	14 (41.2)	
Combination therapy	9 (69.2)	20 (58.8)	
*Antihypertensive drug class, n (% of treated patients)*			
Angiotensin-converting enzyme inhibitor	7 (53.8)	15 (44.1)	—
Angiotensin II receptor blocker	3 (23.1)	15 (44.1)	—
Calcium channel blocker	4 (30.8)	13 (38.2)	—
Diuretic	7 (53.8)	14 (41.2)	—
Beta-blocker	1 (7.7)	2 (5.9)	—
Alpha-blocker	1 (7.7)	0 (0.0)	—
*Ultrasonographic steatosis grade, n (%)*			0.445
Grade 2	40 (93.0)	85 (90.4)	
Grade 3	3 (7.0)	9 (9.6)	
Fasting plasma glucose, mg/dL	91.42 ± 13.28	91.24 ± 10.16	0.933
Urea, mg/dL	30.0 (26.5–34.5)	30.0 (26.0–35.5)	0.635
Creatinine, mg/dL	0.84 ± 0.15	0.83 ± 0.15	0.948
Sodium, mEq/L	140.0 (138.5–142.0)	140.0 (139–141.8)	0.521
Potassium, mEq/L	4.4 (4.2–4.5)	4.4 (4.2–4.6)	0.852
Albumin, g/L	45.0 (43.5–47.0)	46.0 (44.0–47.0)	0.443
AST, U/L	23.0 (17.5–35.5)	20.0 (15.2–28.0)	0.095
ALT, U/L	40.0 (27.5–61.5)	31.0 (22.0–53.5)	0.073
GGT, U/L	34.0 (26.0–58.5)	28.5 (19.2–44.8)	0.023
Triglycerides, mg/dL	158.0 (119.5–217.0)	154.0 (119–221.2)	0.844
Total cholesterol, mg/dL	195.0 (172.5–208.0)	196.5 (171.2–224)	0.331
LDL cholesterol, mg/dL	113.14 ± 29.79	118.41 ± 30.08	0.341
HDL cholesterol, mg/dL	42.0 (36.0–51.0)	40.0 (35.0–45.8)	0.210

Data are presented as mean ± standard deviation, median (interquartile range), or number (%), according to variable type and distribution. p values were calculated using the independent-samples t-test for normally distributed continuous variables, the Mann–Whitney U test for non-normally distributed continuous variables, and the Pearson chi-square or Fisher’s exact test for categorical variables, as appropriate. Steatosis-grade distribution was presented descriptively because only 12 participants had grade 3 steatosis. Ambulatory hypertension was defined solely according to measured ABPM thresholds, whereas antihypertensive medication use was recorded as a separate baseline variable. AST, aspartate aminotransferase; ALT, alanine aminotransferase; GGT, gamma-glutamyl transferase; LDL, low-density lipoprotein; HDL, high-density lipoprotein.

**Table 3:** Ambulatory blood pressure monitoring parameters according to circadian blood pressure pattern.

Variable	Dipper Pattern (*n* = 43)	Non-Dipper Pattern (*n* = 94)	*p* Value
24-h mean SBP, mmHg	130.0 (118.5–136.0)	134.0 (122.0–145.0)	0.027
24-h mean DBP, mmHg	77.47 ± 8.69	79.16 ± 9.85	0.335
24-h mean heart rate, beats/min	79.30 ± 7.75	75.70 ± 8.97	0.025
Daytime mean SBP, mmHg	135.0 (123.0–142.5)	135.5 (123.2–145.0)	0.505
Daytime mean DBP, mmHg	81.14 ± 8.63	80.51 ± 9.98	0.722
Daytime mean heart rate, beats/min	82.33 ± 8.71	77.84 ± 9.39	0.009
Nighttime mean heart rate, beats/min	71.0 (66.0–76.5)	69.0 (62.2–78.0)	0.445
Nocturnal hypertension, *n* (%)	22 (51.2)	72 (76.6)	0.003
Nocturnal systolic BP decline, %	13.2 (11.7–15.8)	3.7 (0.2–6.0)	—
Nocturnal diastolic BP decline, %	19.2 (15.6–21.3)	7.4 (3.4–10.6)	—

Data are presented as mean ± standard deviation or median (interquartile range), according to distribution. Nocturnal hypertension was defined as nighttime mean SBP ≥ 120 mmHg and/or DBP ≥ 70 mmHg. SBP, systolic blood pressure; DBP, diastolic blood pressure.

**Table 4:** Multivariable logistic regression analysis of factors associated with non-dipping blood pressure pattern.

Variable	OR	95% CI	*p* Value
Age, per 1-year increase	1.012	0.978–1.047	0.501
Male sex	0.697	0.329–1.479	0.348
Antihypertensive treatment	1.045	0.454–2.408	0.917
Grade 3 hepatic steatosis	1.551	0.383–6.285	0.539

The dependent variable was non-dipping status. Reference categories were female sex, absence of antihypertensive treatment, and grade 2 hepatic steatosis. OR, odds ratio; CI, confidence interval.

## Data Availability

The data supporting the findings of this study are available from the corresponding author upon reasonable request, subject to institutional and ethical restrictions.
